# On the performance of fusion based planet-scope and Sentinel-2 data for crop classification using inception inspired deep convolutional neural network

**DOI:** 10.1371/journal.pone.0239746

**Published:** 2020-09-28

**Authors:** Nasru Minallah, Mohsin Tariq, Najam Aziz, Waleed Khan, Atiq ur Rehman, Samir Brahim Belhaouari

**Affiliations:** 1 Department of Computer Systems Engineering, University of Engineering and Technology Peshawar, Peshawar, KP, Pakistan; 2 National Center for Big Data and Cloud Computing (NCBC), University of Engineering and Technology (UET) Peshawar, Peshawar, KP, Pakistan; 3 ICT Division, College of Science and Engineering, Hamad Bin Khalifa University, Doha, Qatar; Politechnika Slaska, POLAND

## Abstract

This research work aims to develop a deep learning-based crop classification framework for remotely sensed time series data. *Tobacco* is a major revenue generating crop of Khyber Pakhtunkhwa (KP) province of Pakistan, with over 90% of the country’s *Tobacco* production. In order to analyze the performance of the developed classification framework, a pilot sub-region named Yar Hussain is selected for experimentation work. Yar Hussain is a tehsil of district Swabi, within KP province of Pakistan, having highest contribution to the gross production of the KP *Tobacco* crop. KP generally consists of a diverse crop land with different varieties of vegetation, having similar phenology which makes crop classification a challenging task. In this study, a temporal convolutional neural network (TempCNNs) model is implemented for crop classification, while considering remotely sensed imagery of the selected pilot region with specific focus on the *Tobacco* crop. In order to improve the performance of the proposed classification framework, instead of using the prevailing concept of utilizing a single satellite imagery, both Sentinel-2 and Planet-Scope imageries are stacked together to assist in providing more diverse features to the proposed classification framework. Furthermore, instead of using a single date satellite imagery, multiple satellite imageries with respect to the phenological cycle of *Tobacco* crop are temporally stacked together which resulted in a higher temporal resolution of the employed satellite imagery. The developed framework is trained using the ground truth data. The final output is obtained as an outcome of the SoftMax function of the developed model in the form of probabilistic values, for the classification of the selected classes. The proposed deep learning-based crop classification framework, while utilizing multi-satellite temporally stacked imagery resulted in an overall classification accuracy of 98.15%. Furthermore, as the developed classification framework evolved with specific focus on *Tobacco* crop, it resulted in best *Tobacco* crop classification accuracy of 99%.

## Introduction

Seasonality is one of the most important characteristics of vegetation. Multi-temporal remote sensing is an effective source to monitor and observe the growth dynamics for classification of vegetation and to analyze spatio-temporal phenomena (trends and changes) over the time, using time series data [1]. As remote sensing time series data is being generated at a high scale and enormous rate, it is needed to fully utilize its characteristics for effective land-cover classification. Such remote sensing data has rich content of seasonal patterns and its relative sequential association, which can be very beneficial while performing classification [2]. The provision of additional information by the remotely sensed time series data resulted in increasing interest from researchers and academia in processing of time series data, to extract features for retrieving useful information about the conditions and vegetational growth patterns [3]. While existing approaches to temporal feature extraction offer various ways to represent vegetation dynamics, however in reality, finding an effective and appropriate practical approach is not an easy task [4]. Artificial neural networks inspired from human biological learning systems are widely used machine learning algorithms. They have shown promising performance in diverse fields such as automatic decision support in medical diagnosis and classification of archaeological artifacts [5, 6]. In [7] the use of temporal features for *Tobacco* crop estimation and detection using feed forward neural network resulted in the achievement of more than 95% accuracy.

Deep learning models, or Deep Artificial Neural Network (ANN) with more than two hidden layers, have ample model sophistication to learn from end-to-end data representations rather than manual feature engineering, based on human experience and knowledge [8, 9]. Deep learning has been seen in recent years as a breakthrough technology in machine learning, data mining and remote sensing science [10]. Due to the versatility of deep learning models, expert free automated learning, computational efficiency, and feature representation studies specially related to Image classification hugely takes advantage of Deep learning [11]. Crop classification by Deep CNN shows improved performance in comparison to traditional machine learning methods [12]. A comprehensive study has been conducted to evaluate the performance of a temporal deep convolutional model (TempCNN), while using the satellites time series data [13]. They compared the performance of TempCNN to a traditional machine learning algorithm, Random Forest and a deep learning approach Recurrent Neural Network (RNN), which is suitable for temporal data. Their result shows that TempCNN is more accurate in classifying the satellite time series data than the other state of the art approaches. For deep learning-based studies of different vegetation, a novel domain specific dataset, CropDeep is introduced in [13]. The images in the dataset is collected by different cameras, IoT devices and other equipment. Further, different deep learning models are applied and compared their performance. They concluded that although deep learning algorithms have significant performance while classifying different crops. their is still room for improvement of the algorithms. A transformer architecture for embedding time-sequences is adapted in [14, 15], in order to exploit the temporal dimension of time series data. They proposed the replacement of convolutional layer with encoders that operate on unordered sets of pixels to exploit the courser resolution of the satellite images publically available. Their method comes up with the decreased processing time and memory requirement and also with improved precision. Another deep learning approach, the long short term memory(LSTM) network, is employed to utilize the temporal characteristics of time series data for land cover classification task [16]. The LSTM network originated from text and speech generation is employed to earth observation. They compared its performance with Support Vector Machine (SVM), RNN and classical non-temporal CNN and achieved state of the art performance. The advantage of CNNs compared to traditional machine learning technique is evident from [17, 18], where independent training of random forest, a traditional machine learning algorithm and CNN, a deep learning algorithm is studied and its concluded that the performance of CNN is better in terms of speed as well as accuracy. Furthermore, in [19], the effectiveness of the Conv-1D model in classifying the crop time series temporal data representation is studied and found efficient.

CNNs have become the defacto standard for various machine learning operations especially, studies related to image classification during the past decade. Deep CNN with multiple hidden layers and millions of parameters have the ability to learn complex patterns and objects, provided if trained appropriately on a massive size dataset with ground truth labels. With proper training, this ability of complex learning makes them a feasible tool in different machine learning applications for 2D signals such as images and video frames.

## Related work

Our proposed study is a pixel based classification model that takes spectral and temporal features into consideration. Several studies have been conducted in the domain mentioned in the previous section, that used TempCNNs and RNNs. The most relevant and comparable to our study is conducted by C. Pelletier et al [13]. They have done an exhaustive study to prove TempCNNs as one of the best candidates for classification of crop types using multi temporal satellite imagery. Red, Green and NIR spectral bands, and some spectral indices (IB, NDVI and NDWI) has been used as input to the CNN, whereas 13 different classes were predicted as output by the network. Their model achieved an Overall Accuracy of 93.5%, by experimenting on several different hyper-parameters such as model depth, batch size and number of bands. In comparison, our model utilizes temporal inception blocks as well as a fusion of multi satellite imagery that helps learn richer features and hence results in a much higher overall accuracy of 98.1%.

## Data and methodology

### A. Study area

**Fig 1:**
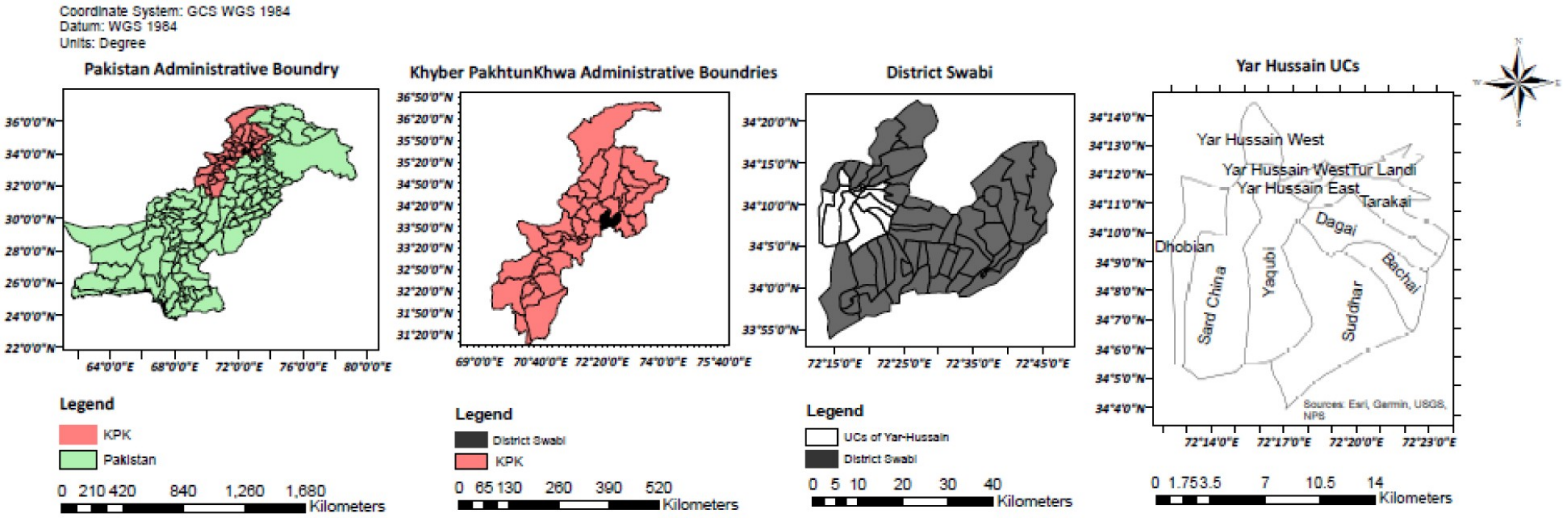
Locality map of region of interest.

Our study area is located in the Khyber Pakhtunkhwa (KP) province of Pakistan. More specifically, for our experimentation work in KP province we selected the Yar Hussain tehsil of District Swabi as presented in Fig 1. This area has wide arable land and a diverse vegetational environment. This region is known for the maximum growth of quality *Tobacco* crop, and has great revenue generation potential for KP province in-terms of taxable income.

### Remote sensing multi-spectral data

In our experimental setup, we utilized the following two types of remotely sensed data. This data comprise of Sentinel-2 and Planet-Scope.

#### Sentinel-2

It’s an open data satellite imagery, acquired from Copernicus open hub Sentinel-2 satellite [20]. Sentinel-2 is a Copernicus Program earth observation mission which systematically acquires optical imagery over land and coastal *Water*s at a high spatial resolution (10m to 60m). In our experimental study with focus on *Tobacco* crop classification, we considered remotely sensed imagery of our pilot region, acquired on 5th, 11th, 26th and 31st of May 2019, while keeping in view the phenological cycle of *Tobacco* crop.

#### Planet-Scope

**Fig 2:**
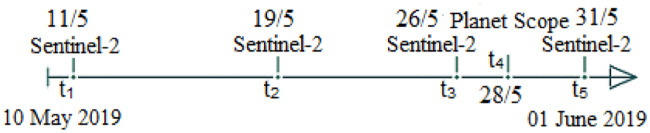
Timeline of the acquired images of the regions of interest.

The Planet-Scope constellation, with 120 satellites currently in orbit, makes up the largest commercial satellite fleet in history, collecting daily images of the entire landmass of Earth [21]. Its sensors are capable of capturing four different multispectral bands—including red, green, blue, and near-infrared multispectral bands, with resolution of 3-5 meters, which is reasonable to analyze and track changes in vegetation and forest cover. Planet-Scope is a a commercial satellite whose data can be purchased from Planet INC [22]. In our experimental setup, Planet-Scope [22] imagery of the pilot region, acquired on 27th of May, 2019 is considered. Fig 2 shows the timeline of the acquired images of the regions of interest.

### Ground survey for data collection

**Fig 3:**
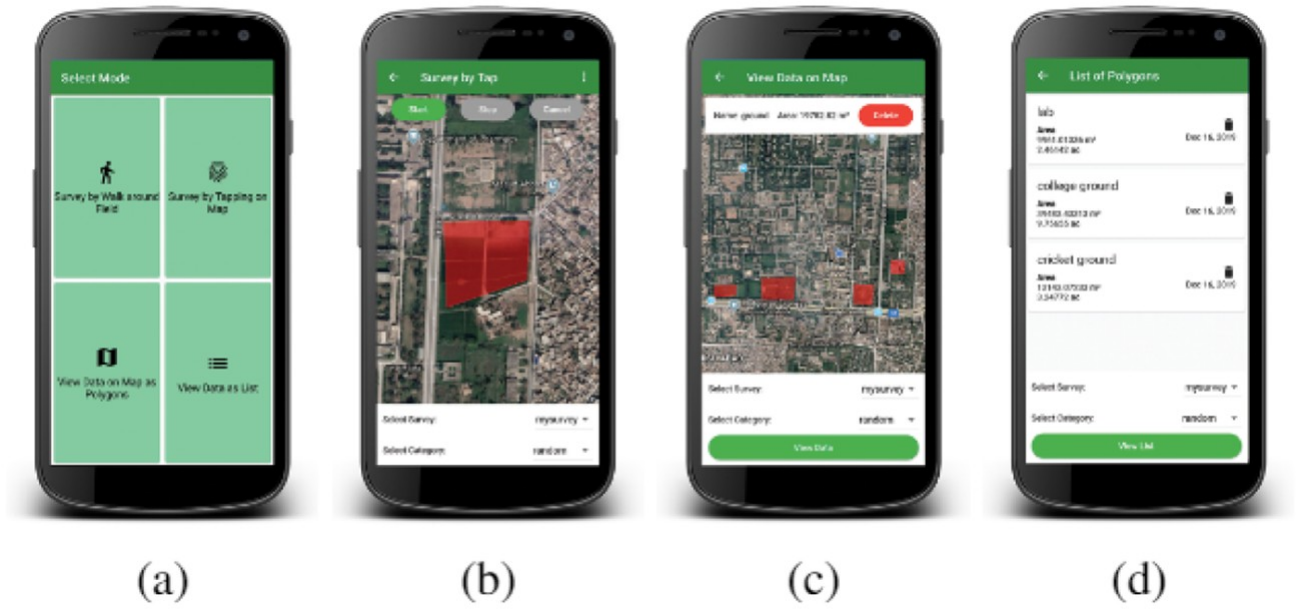
(a). GEOSurvey selection modes. (b). Drawing a polygon by tapping on google map. (c). Viewing the survey data on map. (d). View data as a list.

The ground data collection surveys of the pilot region were conducted using an indigenously developed Geo Survey application [23]. A brief overview of the Geo Survey application is pictorially presented in (Fig 3) [7]. (Fig 3(a)) presents the main view of the application, with multiple choices to choose the method of survey. (Fig 3(b)) shows a polygon drawn around the survey area by choosing the tapping option from the main menu, while (Fig 3(c)) represents viewing of the conducted survey. (Fig 3(d)) presents the data viewing capability of the Geo Survey application. The developed Geo Survey application is native, which is using JAVA programming language. The survey data is being saved in google firebase real time database. Data from firebase is being downloaded in JavaScript Object Notation (JSON) format, and converted into KML using indigenous python scripting. The database used for the storage of data is MySQL. Finally, KML is converted into shapefiles using ARCGIS, for training and testing the performance of the proposed model. With the choice of retrieving a polygon by encircling or by selecting different points interactively, our survey application proved to be cost effective and time efficient, as compared to other traditional methods. In our experimental work, the underlying land cover was divided into five different classes; including *Urban*, *Wheat*, *Tobacco*, *Water* and *Other Vegetables*.

### Data preparation

The acquired remotely sensed imagery of (Fig 2) were further preprocessed using the following steps.

#### Spatial resampling

Due to different band resolutions of both Sentinel-2 and Planet-Scope, spatial re-sampling has been carried out using bi-linear interpolation. While considering the 2x2 neighborhood values of a known pixel, Bi-linear interpolation takes a weighted average of these 4 pixels and calculate its final interpolated value and designates it to the unknown pixel. Tables 1 and 2 shows band designations, spectral and spatial resolutions of Sentinel-2 and Planet-Scope. All bands of Sentinel-2 have been resampled to the resolution of Planet-Scope with a resolution of 3 meters.

**Table 1:** Band composition of Sentinel 2.

Sentinel-2 Bands	Coastal Aerosol	Blue	Green	Red	Red Edge	Red Edge	Red Edge	NIR	Narrow NIR	*Water* Vapor	Cirrus	SWIR	SWIR-2
Wavelength Start Range (nm)	442.7	492.4	559.8	664.6	704.1	740.5	782.8	832.8	864.7	945.1	1373.5	1613.7	2202.4
Wavelength End Range (nm)	442.2	492.1	559	664.9	703.8	739.1	779.7	832.9	864	943.2	1376.9	1610.4	2185.7
Spatial resolution (m)	60	10	10	10	20	20	20	10	20	60	60	20	20

**Table 2:** Band composition of Planet-Scope.

Planet-Scope Bands	Blue	Green	Red	Near Infra-Red (NIR)
Wave length (μm)	0.49	0.55	0.63	0.82
Spatial Resolution (m)	3-5	3-5	3-5	3-5

#### Layer stacking

Normalized Difference Vegetation Index (NDVI), a spectral index was calculated separately for both Sentinel-2 and Planet-Scope satellite imageries. In literature, NDVI is commonly used as input in addition to the acquired spectral bands of the data. This usually helps in handling the non-linearity among the spectral bands of the input data. NDVI is a manually calculated spectral feature, which is quite useful for detecting healthy vegetation and is calculated as follows in Eq (1).

$$\begin{array}{c} NDVI=(NIR-Red)/(NIR+Red) \end{array}$$ (1)

In our experimental setup, NDVI is calculated and layer stacked as an additional layer with the acquired multispectral imagery.

#### Temporal stacking

The final imagery that is used by our proposed model is a temporal stack of both Sentinel-2 and Planet-Scope images for that of the pilot region acquired at various temporal time-stamps, selected with reference to the phenological cycle of *Tobacco* crop, as presented in (Fig 3). More specifically, the resulting temporal stacked imagery consists of 20 overall bands, in which 4 bands are of Planet-Scope, and the remaining 16 are of Sentinel-2 satellite imagery.

### Dataset

The samples set collected during the survey of the pilot region (Table 3) were divided into two subsets, namely training and testing sets. The training and testing data sets have overall 80% and 20% representation of the sample dataset. Additionally, a 15% data of the training set is separated as a validation set. Furthermore, stratified k-fold technique is used with 8 folds for statistical validation and performance analysis of our proposed model. The dataset consists of multi-spectral imagery from 2 different satellites Sentinel-2 and Planet-Scope. Sentinel-2 provides 13 different spectral bands with temporal resolution of 5 days, whereas Planet-Scope provides 4 different spectral bands with 1-day temporal resolution. Spectral bands were selected based on their capability and the extent of provision of the relative information contents with reference to our target research work. Three bands including Red Green and Near InfraRed with 3m and 10m spatial resolution were selected for Planet-Scope and Sentinel-2, respectively. The blue band is discarded because it’s not useful in crop classification and is quite sensitive towards atmospheric particles such as dust and clouds [24]. The number of polygons and pixels, collected through ground survey, for each ground class is presented in Table 1.

**Table 3:** Number of polygons and pixels in classes.

Label	Classes	Polygons	Pixels
***0***	*Other Vegetables*	21	25886
***1***	*Wheat*	94	32814
***2***	*Tobacco*	49	57655
***3***	*Water*	50	4769
***4***	*Urban*	194	7402
	Total	408	128526

## Proposed deep learning model

### Convolutional neural network

**Fig 4:**
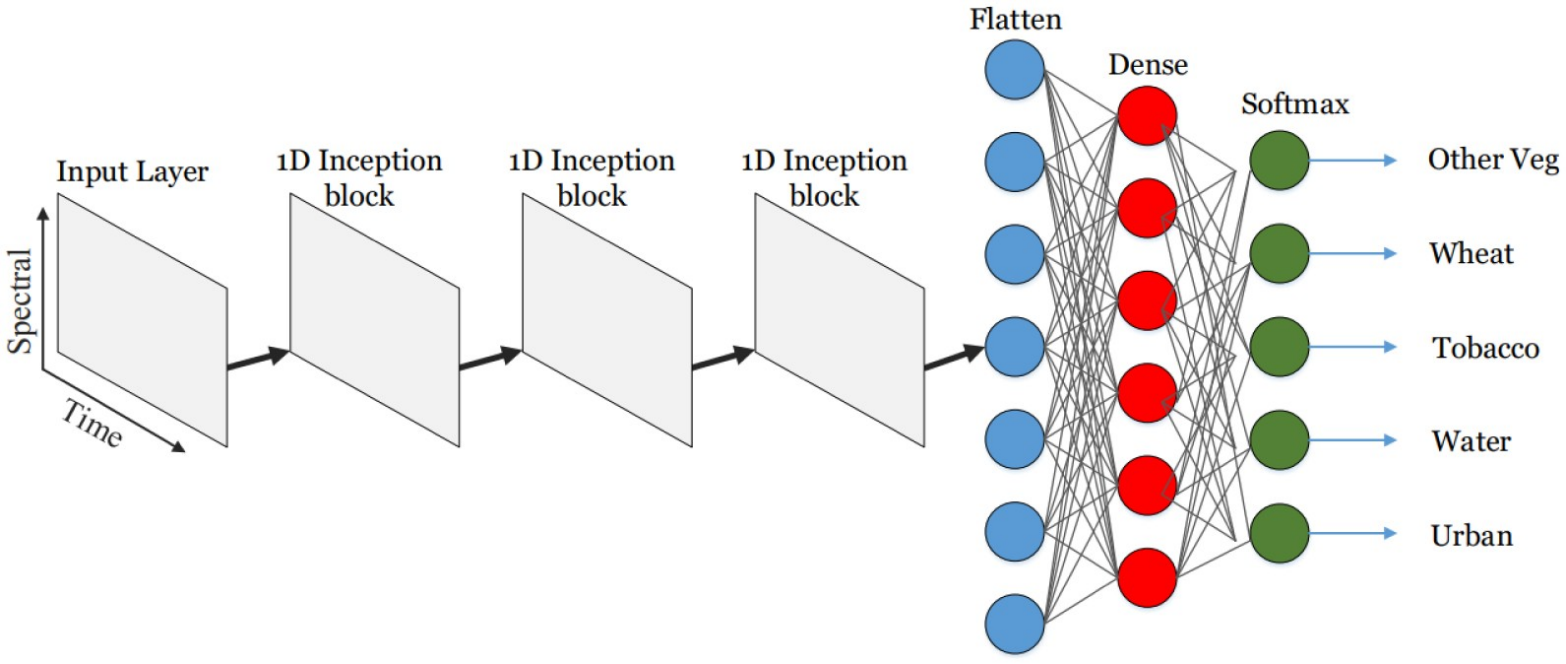
CNN model.

The proposed model shown in Fig 4, is based on the work of [25] and [26]. As discussed in the previous section our experimental setup uses satellite time series imagery, to learn spectro temporal features. Our model consists of three temporal convolutional inception blocks, followed by a Dense layer and a Softmax layer for classification. Each temporal convolutional inception block utilizes filters of size 1,3 and 5 in parallel to provide feature maps that are concatenated and passed to the subsequent layer as shown in Fig 4. Each filter size (1,3 and 5) has 32 filter units which add up to 96 units in a single temporal convolutional inception block. Before filtering, the input is zero padded in order for activation maps to be of the same size and stacked appropriately. These blocks are responsible for learning Spectro temporal features, as 1D convolutional filters are used, which are already proven effective for learning temporal features [27]. Pooling layers are not used in our architectures, as it’s mostly used in computer vision tasks where a whole image or parts of image are provided to the neural network as 2D signals with channels on the third axis, and it is mostly used for robustness against noise and to highlight most dominant spatial features. The proposed model is not using the spatial data, due to the lack of delineation labels, therefore only spectral and temporal data is supplied as input to the network in the form of 2D matrix where x-axis represents the temporal axis and y-axis represents the spectral axis. Therefore, pooling will only result in dimensionality reduction and in turn decrease in accuracy as discussed in [28]. The implemented model is a pixel-based classification algorithm, it takes a pixel as input, and outputs the predicted class that it belongs to. Each pixel represents the spectral reflectance of the on-ground land cover class. The spectral reflectance may vary with the change in time, lighting conditions and other atmospheric factors. Similarly, Vegetations have phenological cycles, and the state of crop is subject to change during that cycle, which in turn contributes towards changes in its spectral reflectance.

**Fig 5:**
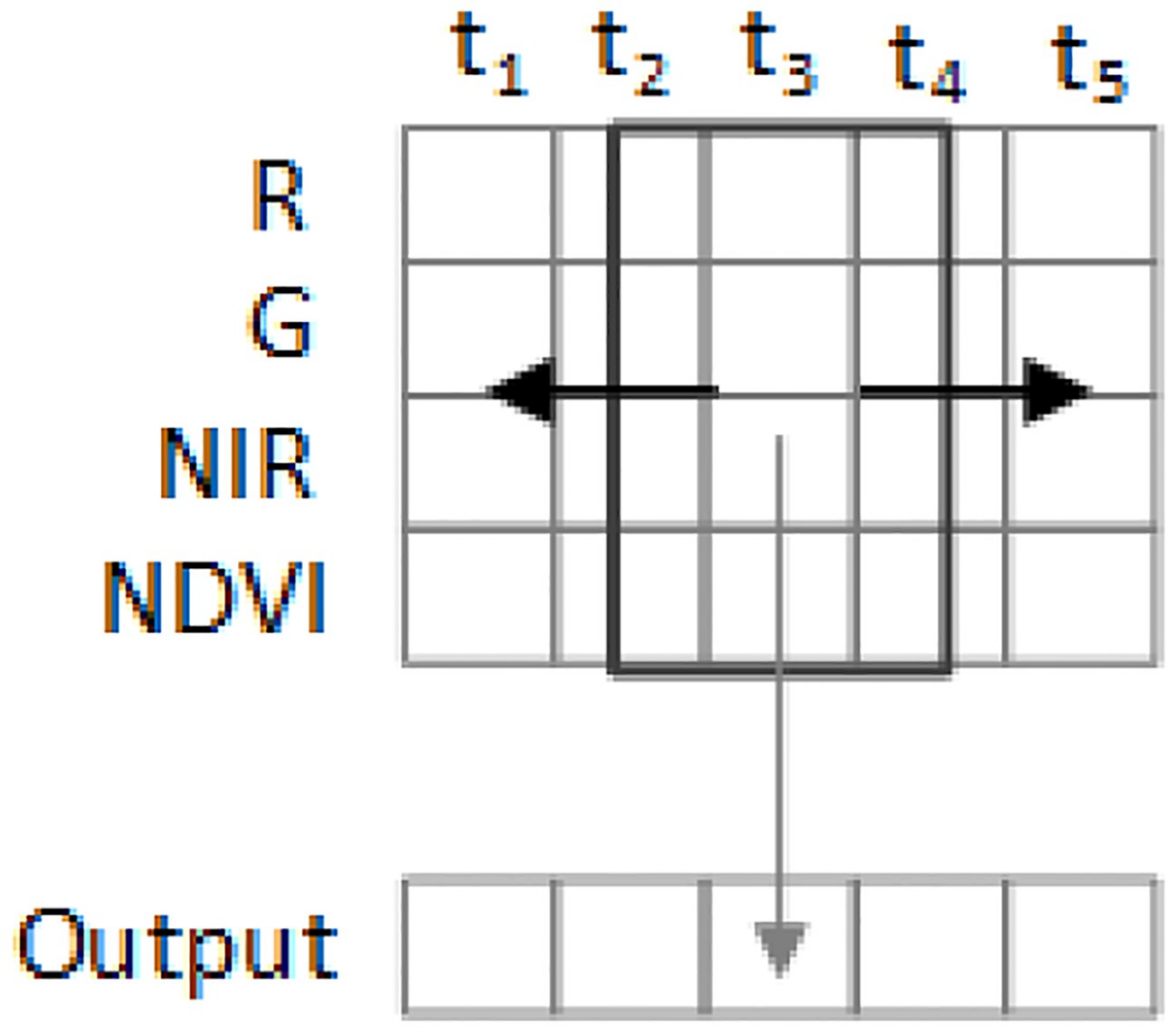
Structure of input data.

The Input to our neural network is 4x5-2D matrix as presented in (Fig 5), where a row represents spectral bands while the columns represent timesteps (image acquisition dates), as presented in (Fig 2). So, the same pixel of different dates is stacked in columns of the matrix. In (Fig 5) the structure of the input is discussed where Red, Green, Near Infra-Red and Normalized Difference Vegetation Index data are denoted by R, G, NIR, NDVI respectively, as the row labels, whereas timesteps are denoted by t1, t2, t3 … t5.

### Inception 1-D convolutional block

**Fig 6:**
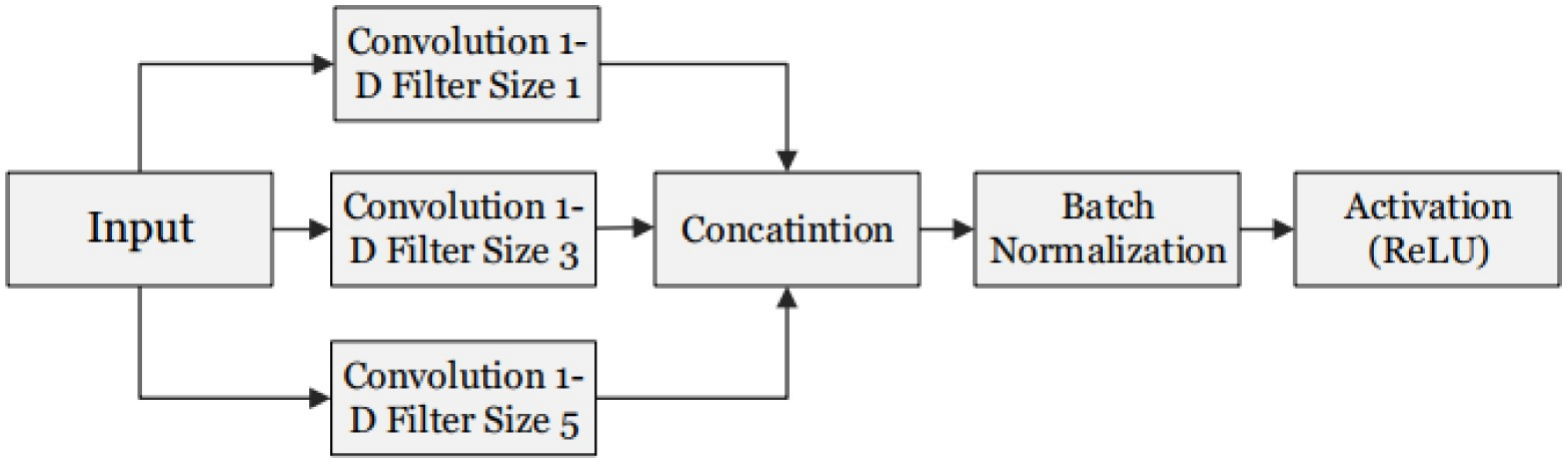
Inception-1D convolution block.

The inception block of our model architecture is one dimensional convolution and is shown in (Fig 6). It runs from left to right (on temporal axis) as can be seen in Fig 5, hence the term temporal convolution is used. The intuition behind it is that they will learn the spectro-temporal features of the crops. The filter size 3 learns the changes in 3 timesteps, filter size 5 learns in 5 timesteps and filter size 1 will learn in a single time-step. These three parallel convolutions are the novelty we added to our model, inspired by the famous inception net, and are giving significantly better results than the same network without an inception block, which gives 92% accuracy. Feature maps obtained from the 1-D convolutions are stacked (concatenated) and passed on to the batch normalization layer, and finally an activation function is applied to introduce non-linearity into our model. As neural networks are prone to overfitting [29], several regularization techniques are employed to curb it. Dropout regularization is used after each block with the percentage set to 20%, which helps reduce overfitting by disabling a few features from each layer during training. L2 regularization of scale 10-6 is utilized, which is proven to be very effective as it settles for a less complicated model by introducing a penalty. Number of epochs is set to 15. Furthermore, early stopping is used with patience of zero validation loss.

### Model loss and accuracy

**Fig 7:**
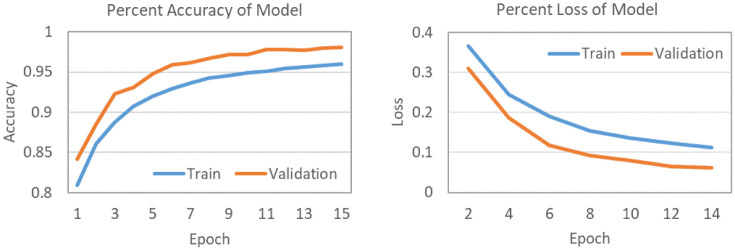
Model accuracy and model loss.

**Fig 8:**
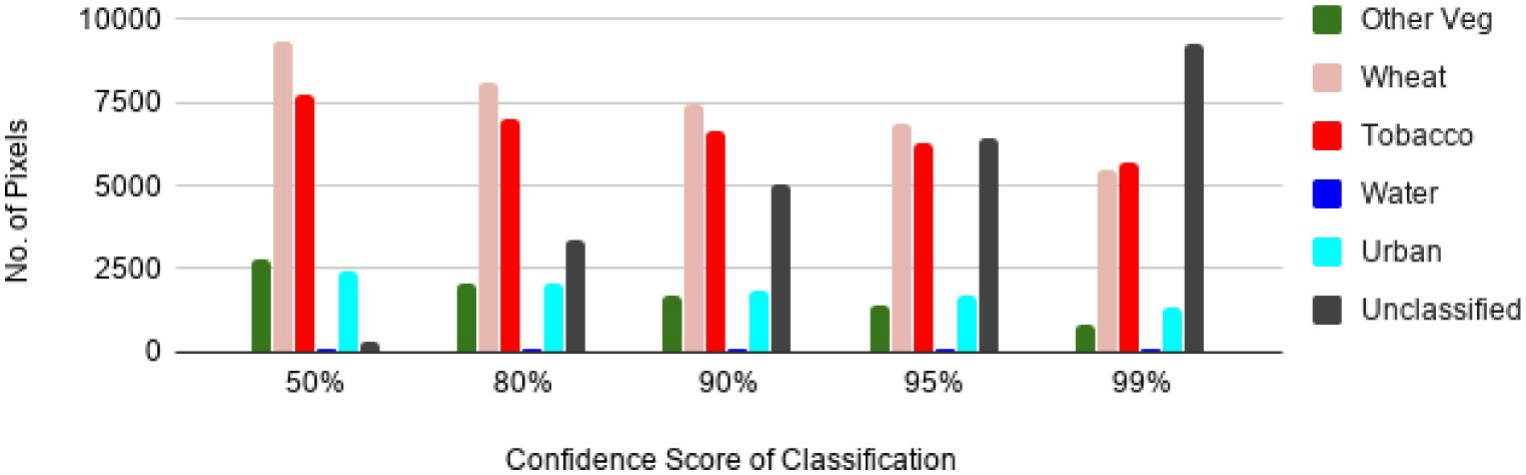
Pixel count of all classes in Yar Hussain regions with confidence score.

In this section, we discuss the models training and validation accuracy and loss throughout the training phase. Fig 7 shows the training and validation accuracy of the base model which demonstrates the logarithmic curve for both sets. An important thing to notice is that both curves follow the same pattern and keep increasing till our epoch limit that is set to 15. Similarly, model’s loss for both sets is depicted in Fig 7, which follows a smooth positive decay curve without any significant fluctuations, and the loss is converging towards the end of the curve. The reason that test set accuracy is more than train set is that we are using a drop-out regularization in training which disables some of the features (it’s given in percentage) in each layer to keep it from overfitting. Additionally, the use of early stopping overcomes the overfitting problem of the model. In order to analyze the performance of the proposed model and to investigate its trend in classification performance, the classification results were generated with 50%, 80%, 90%, 95% and 99% confidence score, as presented in Fig 8. Fig 8, present the number of pixels of the classified classes of the pilot region with their confidence scores. It can be observed from Fig 8 that increasing the classifier’s confidence threshold results in reduction in number of pixels classified to a specific land cover class and increase in number of unclassified pixels. Similar performance trends were observed for all the considered land cover classes, including *Wheat*, *Tobacco*, *Water*, *Urban* and *Other Vegetables*. Furthermore, it can be observed from the results presented in Fig 8 that an increase in the confidence threshold of the proposed model results in gradual increase in the number of unclassified pixels. This is due to the fact that while considering a high confidence threshold for the classification model, pixels with lower classification probabilities are flagged as unclassified, in order to achieve highly precise results. With the visual inspection on tools like ENVI (Environment for Visualizing Images) [30], it was concluded that a threshold of 90% gives very accurate shape boundaries for different crop fields and *Water* streams etc. Furthermore, it is also observed that the increase in the classification confidence threshold also reduces any excessive outliers, where the algorithm is not really sure of the land cover class. A zoomed in visual analysis is explained in the section of Visual Analysis.

## Classification performance

This section presents parameters on which classification performance has been evaluated [7] and classification report of the proposed model over the pilot region.

### Accuracy assessment parameters

- **Precision**—The precision is termed as user accuracy in land use land cover (LULC) classification, which calculates the presence of correctly predicted number of training data pixels of a class in the classified image. The term is also regarded as precision.
  $$\begin{array}{c} Precision=\frac{\left(Correctly\;classified\;sites\right)}{\left(Total\;number\;of\;classified\;sites\right)} \end{array}$$ (2)
- **Recall (Sensitivity)** Recall or sensitivity can be defined as the number of correctly predicted training data pixels of a class, compared to the total training data pixels provided to the system.
  $$\begin{array}{c} Recall=\frac{\left(Correctly\;classified\;Ground\;Truth\;Sites\right)}{\left(Total\;number\;of\;Ground\;Truth\;sites\right)} \end{array}$$ (3)
- **F1 score**—The weighted average of precision and recall is called F1-score.
  $$\begin{array}{c} F1Score=2*\frac{\left(Recall*Precision\right)}{\left(Recall+Precision\right)} \end{array}$$ (4)
- **Overall-Accuracy**—Overall accuracy is the ratio of the sum of all correctly classified training data pixels to the total number of training data pixels. Where;
  $$\begin{array}{c} Total\;accuracy=\frac{(Number\;of\;all\;correctly\;classified\;samples}{\left(Total\;number\;of\;samples\right)}\times 100 \end{array}$$ (5)

### Classification report

Our experimental work was concluded while employing the best model architecture. More Specifically, in our classification model we utilized batch size 128 and relu as activation function for all the hidden layers. Furthermore, it is observed that the input consisting of only temporally stacked multispectral imagery (Sentinel-2 and Planet-Scope) spectral reflectances without vegetation indices (NDVI) results in best classification performance. The proposed model was utilized for land cover classification of the pilot region and results were obtained in the form of a confusion matrix shown in Table 4. It can be observed from the obtained results, presented in Table 4, that our proposed model resulted in convincing classification performance, with an overall accuracy of 98.15%. Both precision and recall for the *Other Vegetables* class has been the recorded as 97%, while for the *Wheat* class, precision and recall of 98% and 97% has been observed, respectively. Moreover, our proposed model showed remarkable classification performance for *Tobacco* class, with precision and recall performance of 99%. *Water* and *Urban* classes has been perfectly classified, giving 100% precision and recall performance.

**Table 4:** Classification report.

Class	Precision	Recall	F1-Score	Support
*Other Vegetables*	0.97	0.97	0.97	5126
*Wheat*	0.98	0.97	0.97	6530
*Tobacco*	0.99	0.99	0.99	11702
*Water*	1	1	1	906
*Urban*	1	1	1	1442
Accuracy			0.98	25706
Macro Avg	0.99	0.99	0.99	25706
Weighted Avg	0.98	0.98	0.98	25706
Classification Accuracy: 0.981522				

### Land cover classification

**Fig 9:**
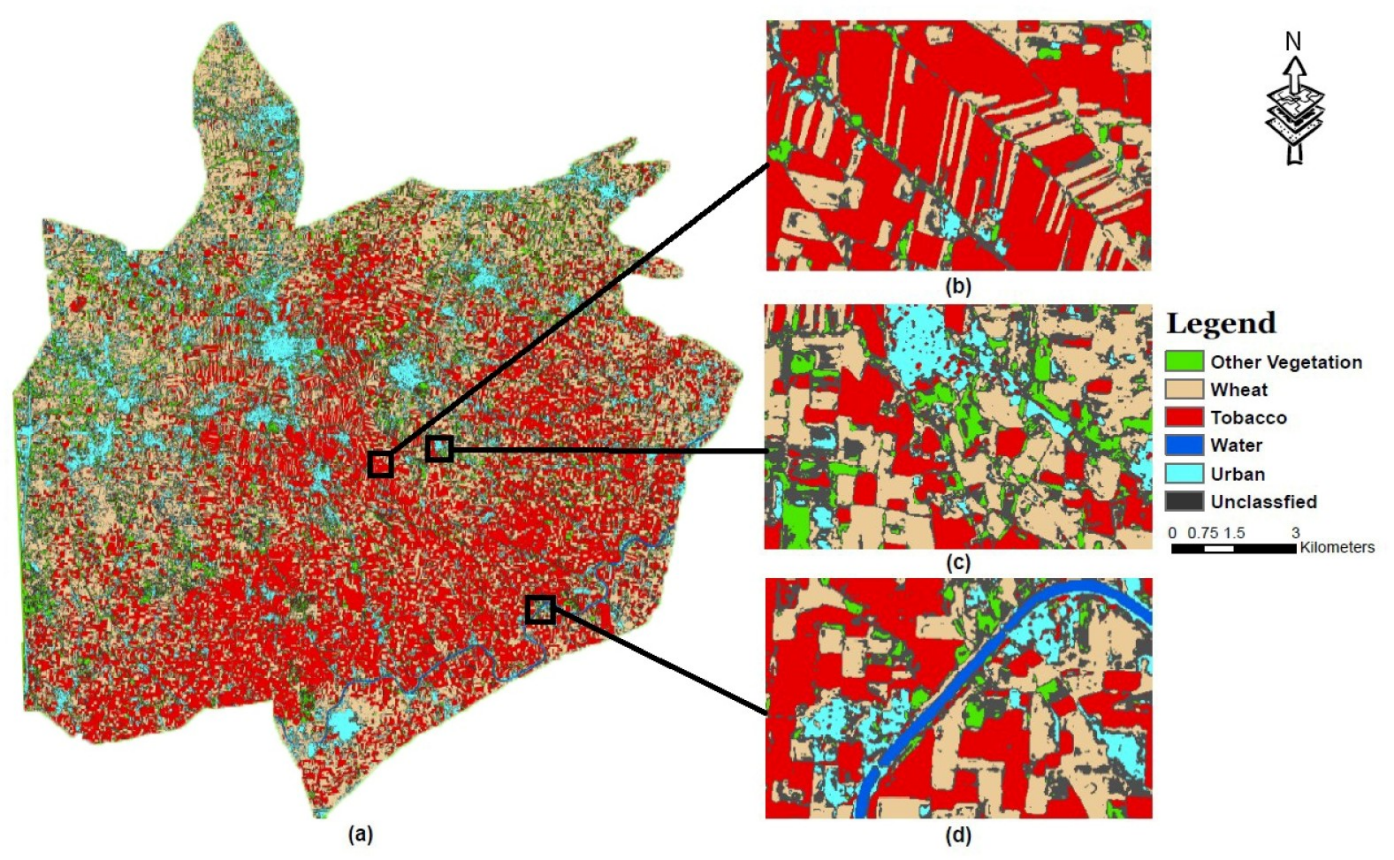
Classified land cover map of Yar Hussain.

Finally, the pilot region of interest was classified by applying our trained Deep Learning model to the temporal stack of raster multispectral remote sensing data and classified landcover map of the pilot region was generated, as shown in Fig 9.

## Results and discussion

In this section we will discuss the quantitative and qualitative outcome of the proposed model as an output of the performed experiments. As Artificial Neural networks do possess a variety of hyper parameters that can be tuned to get the best performance for the task at hand, but trying every possible value for them in combination with others is practically not feasible, as it requires a huge amount of computational power due to the innate compute hungry nature of the algorithm. Therefore, several best practices have been established for certain hyper parameters and can be borrowed for similar tasks from other networks as well. To balance the bias-variance trade off, our network is borrowing parameters such as depth or number of hidden layers; which is set to 3; as well as the number of filter units; which is set to 32; from the work of [13].

As CNNs are computational intensive and performing experiments for finding hyper-parameters requires lots of processing power, therefore we have utilized Microsoft Azure cloud services for the training and experimentation of our model. More details are listed in Table 5.

**Table 5:** Specifications of system used for CNN training.

GPU Model	NVIDIA Tesla k80 GPU
System Memory	56 GB
Number of Processors	1
Number of Cores	6
Processor Model	Intel Xeon 2640V3
Implementation Platform	Keras with Google TensorFlow backend

Our algorithm is using Adam as an optimizer with default learning rate of 10-2, *beta*_1_ of 0.9, *beta*_2_ of 0.999 and epsilon of rate 1*e* − 07, similarly the l2 regularization rate is set to 1.*e* − 6.

We have searched through the other hyper parameters by evaluating them on overall accuracy of training and validation set on 8 folds of data. Experiments were performed with a focus on the impact of the following factors on the land cover classification performance.

- Influence of spectral indices
- Influence of batch size
- Influence of Activation function
- Visual Analysis

### Influence of spectral indices

Neural networks are very powerful algorithms in terms of learning a nonlinear relationship between input and desired output. The depth of neural networks corresponds to the complexity of the features that it learns from the input data. The top layers that are nearer to the input, learn simpler features, whereas the deeper layers learn more complex features. Therefore, neural networks are known to automate the process of feature engineering and shifted the burden towards architecture engineering. However, in remote sensing several spectral indices are calculated from the available electromagnetic spectrum for certain tasks, such as NDVI, NDWI and Birlliance Index etc. for finding green vegetation, and are provided as input feature to classifier, along with the multispectral imagery. Our proposed model has been tested with various combinations of spectral bands and NDVI, to find its impact on the overall classification performance. It can be observed from the results presented in Table 6 that NDVI has very minor impact on the classification performance of the proposed model. This reiterates the fact that ANNs are very powerful in terms of learning compound features from the raw intensities of different spectral bands of pixels, and are intelligent enough to extract meaningful information in the deep layers on its own, instead of explicitly incorporating additional information in the remotely sensed data, such as spectral indices, such as NDVI, which themselves are calculated from the same remotely sensed input data.

**Table 6:** Impact of NDVI on the overall accuracy of the model.

	Train	Valid
Spectral Bands only	95.90±0.16	98.09±0.21
Spectral Bands + NDVI	96.00±0.14	98.19±0.15

### Influence of batch size

Due to the limitation of computational power, a study of the influence of batch size on the overall training and validation accuracy and training time was conducted on the 8 folds of data. Four different batch sizes were used with values 16, 32, 64 and 128. As reflected by the results in Table 7, variations in the batch size has very insignificant influence on the overall training and validation accuracy of the model. More specifically, the overall accuracy is increased by increasing the batch size. Therefore, higher batch size value can be used to speed up the training processes.

**Table 7:** Mean overall training and validation accuracy with one standard deviation and time for a single epoch for different batch sizes.

batch size	Train acc	Valid acc	Time (sec)
16	94.90±0.16	97.65±0.16	53
32	95.16±0.26	97.66±0.24	33
64	95.54±0.28	97.80±0.35	14
128	95.90±0.16	98.09±0.21	8

### Influence of activation function

Experiments were conducted while employing three of the most common activation functions to the proposed base model in order to find out their influence on the model accuracy. The three activation functions are briefly described below. The first activation function is sigmoid, which is most commonly used as a logistic function as well and it outputs values in the range 0-1. It is mathematically represented by the following formula.

$$\begin{array}{c} f\left(x\right)=1/\left(1+e^{x}\right) \end{array}$$ (6)

Second function is called Relu, it takes logits as input and outputs zero for negative values whereas linear for positive values. It is described by the following formula.

$$\begin{array}{c} f(x)=max(0,x) \end{array}$$ (7)

Third function is tangent hyperbolic or tanh, it is similar to sigmoid and gives output values between -1 and 1 [31].

It is evident from the results obtained and presented in Table 8 that ReLU is performing better than tanh and sigmoid. Moreover, ReLU also has lower standard deviation in comparison to tanh and sigmoid.

**Table 8:** Model’s overall accuracy with different activation function.

	Train	Valid
sigmoid	94.52±0.43	97.11±0.43
relu	95.90±0.16	98.09±0.21
tanh	95.89±0.67	97.91±0.79

### Visual analysis

The best settings of various factors, were selected for the proposed model based on the results obtained and presented. Experiments were performed using the proposed model to classify the Yar Hussain region in the Swabi District of KP, Pakistan. The classified images of the pilot region were visually analyzed. Samples of the classified visual are shown in Fig 9(b), 9(c) and 9(d) which are zoomed in view of the sub regions of Fig 9(a). In Fig 9(b), it can be seen that our implemented model has classified the *Tobacco* class with very fine boundaries. Towards the edges a small number of unclassified pixels gives the confidence about the classified *Tobacco* class. Fig 9(c) shows a high number of unclassified pixels, the reason is, the mixing of different classes into one another at class boundaries. Fig 8 clearly shows this trend, as the confusion rises with the increase in confidence score the pixel of *Wheat* class followed by *Tobacco* and *Other Vegetables* get into the unclassified class to be sure about the classified classes. A *Water* stream can also be seen at the bottom right corner of Fig 9(a) as well as Fig 9(d), classified accurately with detail using our algorithm. Despite being a pixel based model, our model has shown promising results of predicting different land cover classes with precise shapes.

## Conclusion

In this paper we presented a state-of-the-art mechanism of inception inspired Deep CNN for *Tobacco* crop classification using remotely sensed time series multispectral data. Instead of the conventional approach of utilizing single satellite imagery, both Sentinel-2 and Planet-Scope imageries are stacked together in order to increase its spectral resolution and to avail maximum reflectance information. The multi-satellite imagery stacking results in an improved classification performance of the proposed model. Furthermore, instead of utilizing a single date imagery, the temporal resolution of the applied multispectral data is improved by stacking multi-date satellite imageries. The concept of multi-date satellite imageries stacking is applied with specific focus on the phonological cycle of *Tobacco* crop. Indigenously developed “GEOSurvey” surveying application was utilized for ground truth data survey. It is concluded with the results obtained that the proposed novel approach of TempCNN consisting of three temporal convolutional inception blocks and a fully connected and a SoftMax layer resulted with overall classification accuracy of 98.15% on satellite time series data. Furthermore, the developed classification framework with particular focus on *Tobacco* crop, resulted in the highest *Tobacco* crop classification accuracy of 99%.
